# Proinflammatory MG-63 cells response infection with *Enterococcus faecalis* cps2 evaluated by the expression of TLR-2, IL-1β, and iNOS mRNA

**DOI:** 10.1186/s13104-017-2740-4

**Published:** 2017-08-11

**Authors:** Boy M. Bachtiar, Endang W. Bachtiar

**Affiliations:** Department of Oral Biology and Oral Science Research Center, Faculty of Dentistry, Universitas Indonesia, Jl. Salemba Raya 4, Jakarta, 10430 Indonesia

**Keywords:** Encapsulated and unencapsulated *E. faecalis* cps2, MG-63, TLR-2, IL-1β and iNOS

## Abstract

**Objective:**

We have previously demonstrated that unencapsulated *Enterococcus faecalis* cps2 inhibits biofilm formation of *Candida albicans*, a fungus commonly found with *E. faecalis* in periapical lesion. In this study, we compared encapsulated and unencapsulated *E. faecalis* cps2 strains relationship with osteoblastic (MG-63) cells, whereas *E. faecalis* ATCC 29212 were used as a reference strain.

**Results:**

The binding capacity of *E. faecalis* to MG-63 cells as shown by each tested strain was comparable, but the unencapsulated strain was less invasive compared to the encapsulated and the reference strains. Moreover, quantitative real time-PCR (qPCR) results showed that infecting unencapsulated *E. faecalis* cps2 is a stronger stimulator for toll like receptor 2 (TLR2) and interleukin-1β (IL-1β) mRNAs, but not for inducible nitric oxide synthase (iNOS) mRNA in osteoblastic cells. In conclusion, the performance of unencapsulated *E. faecalis* cps2 when the bacterium interacts with osteoblastic cells is quite different from that of encapsulated *E. faecalis* cps2 and reference strains. It appears that the unencapsulated strain might contribute to the persistence of the periapical inflammatory response, depending on down-regulation of iNOS mRNA expression.

## Introduction

From all known as *Enterococcus* spp., *Enterococcus faecalis* has been studied for its involvement in periapical inflammation [1, 2]. Among its serotypes, especially serotype C that belong to cps 2 genotype, are virulent bacteria, and their capsular polysaccharides are responsible for inflammatory responses [3, 4]. However, there is a discrepant opinion regarding the possible role of *E. faecalis* cps2 in the pathogenesis of periapical inflammation. A report by Pinheiro [5] shows that the most common isolate that found in infected root canal with periapical lesion is *E. faecalis* cps1, thus unencapsulated strain [3]. This report contradicts our results, where the *E. faecalis* cps2 was found to be the dominant bacterium from endodontic patients, and it consists of two different strains [6]. Both isolates possess all the cps2 genes [7], but the insertion sequence 6770, detected by qPCR [7], may influence the translation of some of the capsule genes and the synthesis of the polysaccharide. How these differences affect phenotype properties of *E. faecalis* serotype C oral isolates when the bacterium interacts with bone-forming cells, remains to be elucidated.

We previously examined the antagonistic interaction between unencapsulated *E. faecalis* cps2 and *Candida albicans* [8], a fungus frequently found together with *E. faecalis* in periapical lesion [9]. The present study aimed to examine the in vitro ability of encapsulated and unencapsulated *E. faecalis* cps2 to infect human MG-63 osteosarcoma cell lines. We also studied the expression of certain inflammatory response-related genes (TLR-2, IL-1β, and iNOS), when the bacteria interact with MG-63 cells.

## Main text

### Methods

#### Adhesion and internalization by MG-63 cells of three *E. facials* strains

**Fig. 1:**
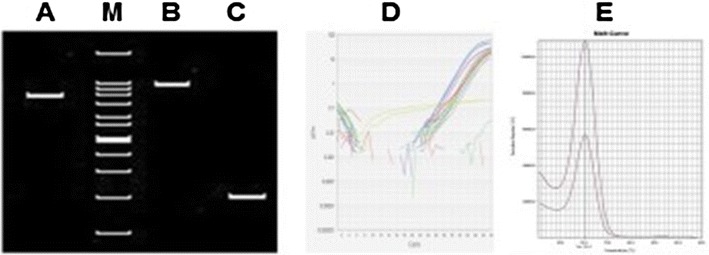
PCR analysis of *E. faecalis* cps types [3] (*left*) and qPCR to identify the presence of IS6770 in cps locus of unencapsulated *E. faecalis* cps2 genome [7]. *A E. faecalis* cps1 (950 bp), *M* DNA marker, *B E. faecalis* cps2 (1098 bp), and *C E. faecalis* cps5 (199 bp). *D* and *E* are representative amplified qPCR and its melting peaks, respectively

*Enterococcus faecalis* strains used in this study were *E. faecalis* cps2 oral isolates. These strains were isolated from endodontic patients in our previous study [6], and were separated in encapsulated and unencapsulated strains using qPCR (Fig. 1). The bacteria had been previously categorized as highly biofilm formation strains [6], while the reference strain (*E. faecalis* ATCC 29212) was included in parallel for the comparison experiment.

The bacteria were maintained and prepared for experiment as previously reported [8], while a modified gentamicin protection assay was performed to study bacteria-host interactions [10]. MG-63 cells were used as host cell in this study and were cultured in DMEM and supplemented with 10% FBS and penicillin, streptomycin, and glutamine. The cells were further stimulated with different *E. faecalis* strains at a Multiplicity of Infection (MOI) of 1000. At different times from the post-infection (3 and 12 h), the RNA from infected MG-63 cells were extracted for mRNA transcription analysis of the targeted inflammatory-related genes. The assays were conducted in duplicate and repeated independently three times, while cells without added *E. faecalis* were used as control. For statistical analysis, Student’s *t* test was performed with Microsoft Excel software. A p value of <0.05 was defined significant.

#### Quantitation of TLR2, IL-1β, and iNOS transcripts from MG-63 cells by real-time PCR

After infection, the total cellular RNA was extracted using Trizol reagent (Invitrogen) followed by reverse transcription using the TaqMan Reverse Transcription kit (Applied Biosystems). The resulting cDNA was amplified by qPCR with specific primers as shown in Table 1. The qPCR analysis was performed in ABI StepOnePlus Real-Time PCR Systems with SYBR Green PCR master mix (Applied Biosystems) according to manufacturer’s protocol. The PCR conditions were set as follow pre-denaturation at 95 °C for 5 min followed by 40 cycles of 95 °C for 10 s, 60 °C for 30 s, and 72 °C for 30 s, and a final extension at 72 °C for 5 min. The melt curve profile was set as follow 95 °C for 15 s, 60 °C for 60 s, and 95 °C for 15 s.

**Table 1:** Primers used for real-time PCR in this study

Primer name	Sequences	References
TLR2	Forward: 5′-ggccagcaaattacctgtgtg-3′ Reverse: 5′-aggcggacatcctgaacct-3′	[28]
IL-1β	Forward: 5′-acgatgcacctgtacgatca-3′ Reverse: 5′-tctttcaacacgcaggacag-3′	[29]
iNOS	Forward: 5′-tctccgaccaccactacagcaa-3′ Reverse: 5′-ggggaactgggcagactcaa-3′	[30]
GDPH	Forward: 5′-aatggaaatcccatcaccatct-3′ Reverse: 5′-cagcatcgccccacttg-3′	[31]
IS6770	Forward: 5′-gatgttgtccgttgtaattgg-3′ Reverse: 5′-ccacatttcttgcgtgtcc-3′	[7]

In this study, the target gene expression was normalized to the level of d-glyceraldehyde-3 phosphate dehydrogenase (GAPDH), and cells without exposure to *E. faecalis* were set to be the control. The formula of fold change 2^−ΔΔCt^ was used to analyze the mRNA expression level of targeted genes [11].

The experiment procedure was performed in triplicate for each sample and repeated two times in separated occasion. The collected data were expressed as mean standard deviation, and the mean quantitative gene expression was compared via Student’s *t* test using Microsoft Excel software. A p level of <0.05 was defined significant.

## Results

### Infection capacity among the three strains

**Fig. 2:**
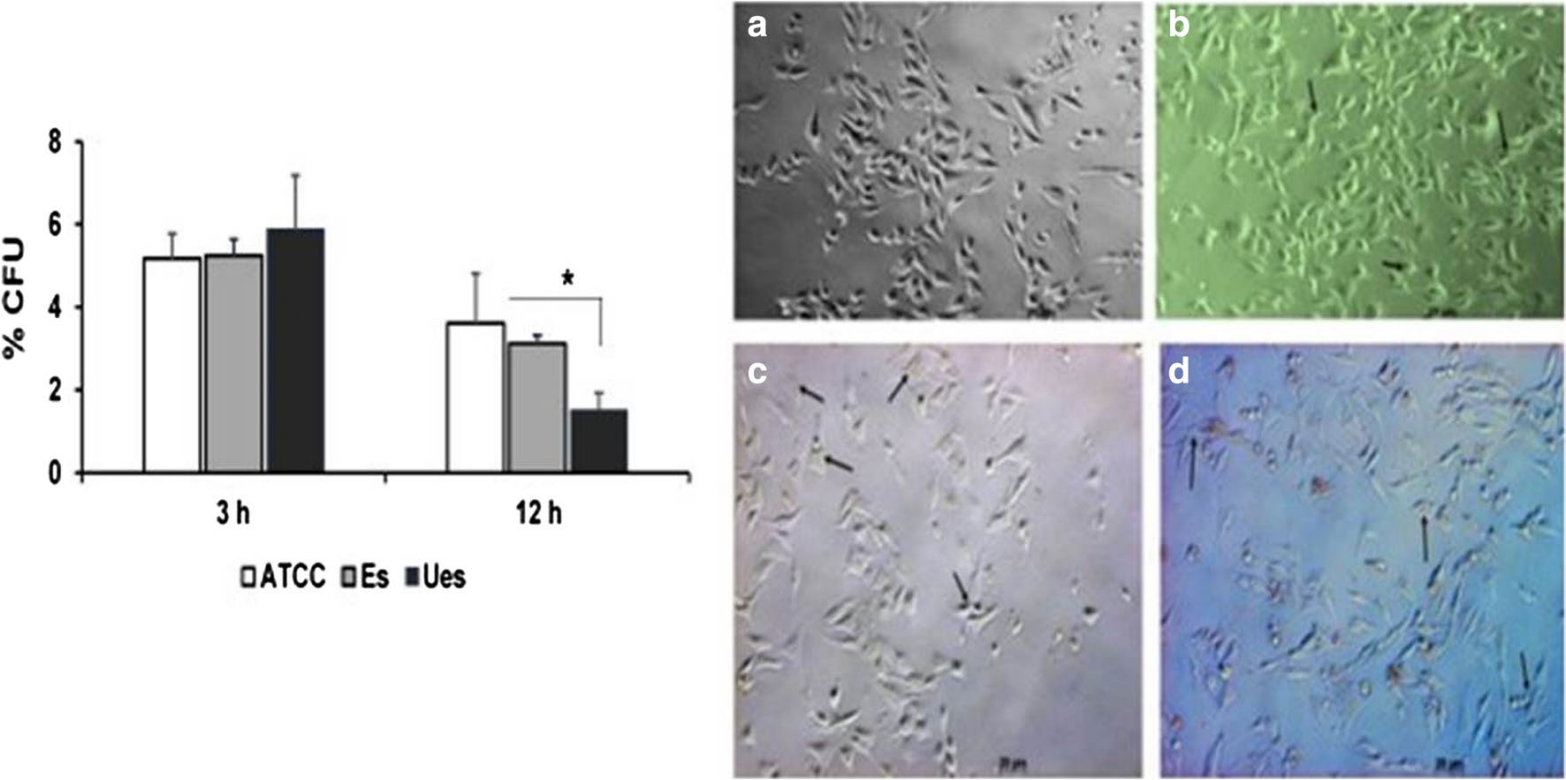
Infection MG-63 cells by tested *E. faecalis* cps2 oral isolates. The binding capacity of encapsulated and unencapsulated strains was comparable after 3 h, but viable inside the cells at least 12 h post infection as assessed by CFU. *p < 0.05. The *right panel* shows the cells that was treated with each *E. faecalis* strain at MOI of 1000 for 3 and 12 h and visualized by using light microscopic images at ×400 magnification. *Bar* 20 µm. **a** Control uninfected cells. **b** Adhesion phase (3 h). **c** and **d** are cells infected with encapsulated and unencapsulated *E. faecalis* cps2, respectively. The *arrows* show adhered and internalized bacteria

*Enterococcus faecalis* strains were tested for their capacity to infect MG-63 cells. Overall, *E. faecalis* strains exhibit a similar adhesion ability to MG-63 cells, and the morphology of infected cells was relatively unchanged during the 3 h time period (Fig. 2). However, the ability of unencapsulated strain to persist in MG-63 cells was impaired, at least for 12 h, compared to the other strains tested (p < 0.05). At this time point, the intracellular bacteria appear to alter MG-63 cell morphology (Fig. 2).

### Different effects of *E. faecalis* strains tested on the expression of TLR-2, Il-1β, and iNOS in MG-63 cells

**Fig. 3:**
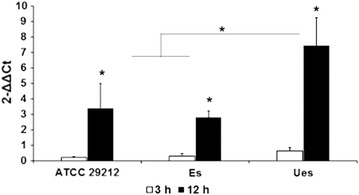
Analysis of TLR2 mRNA expression in MG-63 cells after infected by *E. faecalis* ATCC 29212, encapsulated (Es), and uencapsulated (Ues) strains. Expression of TLR2 mRNA was determined by qPR at 3 and 12 h time points, and normalized to housekeeping gene, GDPH. *p < 0.05

**Fig. 4:**
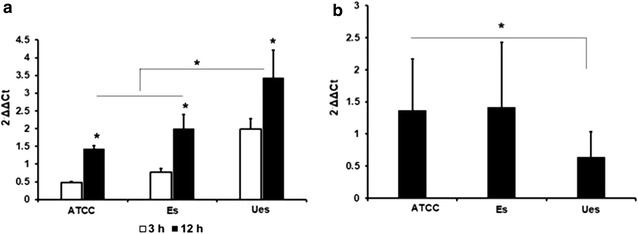
Measurement of IL-1β and iNOS mRNAs expression in infected MG-63 cells. Level of IL-1β mRNA increased after 3 h and remained elicite after 12 h post infection. The unencapsulated (Ues) strain showed a stronger stimulator for IL-1β mRNA expression (**a**), but weakly inducer for iNOS mRNA expression, compared to encapsulated (Es) and ATCC strains, respectively (**b**). The *asterisks* indicate a significant difference

We further determined whether the capacity of the bacterium tested in triggering osteoblast immune response took place through the regulation of certain innate immunity-related genes (*TLR2*, *IL*-*1β*, and *iNOS*). As shown in Fig. 3, at 3 h co-cultured, no significant difference was observed in the expression level of TLR2 mRNA. In contrast, after the 12 h time point, the expression of TLR2 mRNA was increased compared to control (cultured cells without added bacterium), by approximately threefold and sevenfold higher in MG-63 cells co-cultured with encapsulated or reference strain and unencapsulated strains, respectively. Our data also showed an increase in IL-1β mRNA transcription throughout the observation periods (3 and 12 h). On the contrary, the iNOS mRNA expression was only measured after 12 h period, where the internalized unencapsulated strain significantly reduced the transcription level of iNOS mRNA, compared to other tested strains (Fig. 4 a, b).

## Discussion

*Enterococcus faecalis* has been described as a periapical lesion-related species post endodontic treatment [12, 13]. In this in vitro study, the two *E. faecalis* isolates used were serotype C, genotype2. They possess the cps2 genes, but the insertion sequence (IS6770) affects translation of some of the capsule genes and synthesis of the polysaccharide [7]. To answer whether the same serotype C of *E. faecalis*, which displays significant variation in CPS locus may affect their phenotype properties in bacteria–bone cells interaction, we infected osteoblastic MG-63 cells with each oral isolate of *E. faecalis* cps2, and compared the amounts of adherent and internalized bacteria recovered after 3 and 12 h time periods.

When tested individually, all *E. faecalis* strains were attached on MG-63 cell during adhesion phase (3 h) in a similar level. This result indicates that the binding capacity is not strains dependent. It seems that the bacteria retained their capacity to grow as biofilms [6], and subsequently promoted their adherence [14] which is required in the initial step of *E. faecalis* to infect its host cells [15]. Although CPS has a role in modulating the interaction between bacteria and their host cells [10, 16], we assumed that the expression of CPS in *E. faecalis* cps2 does not have a crucial role in the adherence process. However, since the capsule is protective [4], it may interfere with the cell sensing of pathogens that avoid the release of pathogen-associated molecular patterns (PAMPs) signals. On the contrary, the unencapsulated *E. faecalis* cps2 needs to protect itself from the cell defense mechanism. As shown in this study, although the number was lower, the bacterium was still viable after 12 h time period (Fig. 2). The result of this experiment may suggest that the survival of the unencapsulated *E. faecalis* cps2 within bone-cells is crucial, as it reflects the tolerance response of cells to the bacterium and the interaction between them with low spread and slower growth.

To evaluate whether invasive potential differences between all the tested bacteria to cause varied stimulation effects on proinflammatory response, we further compared the expression level of TLR-2, Il-1β, and iNOS mRNAs during bacterial infection the MG-63 cells. The qPCR result demonstrated that the inflammatory effect by *E. faecalis* cps2 was in general a time dependent. When adhesion phase (3 h) in host–bacteria interactions was evaluated, all tested *E. faecalis* strains showed a similar level in up-regulation of the expression of TLR2 mRNA. Interestingly, when the encapsulated strain persisted for 12 h in MG-63 cells, the cells cannot mount a TLR-2 response, as the bacterium might be hidden by the capsule. In contrast, the unencapsulated strain produces a higher induction of TLR2 gene. In addition, the immune response of cells as shown by IL-1β gene was highly induced, even at 3 h (Figs. 3, 4a). This observation suggests that although the unencapsulated strain has a low capacity to invade osteoblast cells, it is still implicated as a pre-requisite for inflammatory bone-cells response by TLR2 activation in osteoblast [17]. This finding may explain the variations in CPS phenotype between isolates of the same serotype C, indicating that factors other than CPS, such as PAMPs, also affect the bacteria invasiveness [18, 19].

According to literature, up-regulated TLR2 initiates a NF-kB signaling cascade that results in a production of proinflammatory cytokines [20, 21]. This study showed that bacterial adhesion per se, shown by either tested *E. faecalis* strains, could be a sufficient stimulus for the expression of IL-1β mRNA, a potent proinflammatory mediator [22]. This is because the expression of IL-1β mRNA in MG-63 cells, by each tested bacterium, was initiated at adhesion time period (3 h) and continued to increase until 12 h post infection (Fig. 4a). Surprisingly, although the IL-1β expression was strongly upregulated by the invading unencapsulated *E. faecalis* cps2, the iNOS mRNA was weakly expressed. If the transcription levels were positively associated with protein production, the current study might suggest that the unencapsulated *E. faecalis* cps2 strains are not a sufficient stimulator for iNOS production as proinflammatory-related enzyme by MG-63 cell lines.

As reported previously, activation of iNOS pathway by cytokines stimulates nitric oxide (NO) production. NO is an important antimicrobial that play is a major role in innate defense mechanism of host cells [23], to pathogens. This study indicates that low level of NO may imply a lower antibacterial activity for different *E. faecalis* strains.

Some studies report the role of NO in inhibiting bone resorption, thus enhancing osteoblast function [24, 25]. However, other studies have shown a controversial result, as NO enhances bone resorption induced by cytokines [26, 27]. Our experiment showed that the very low level of induction of iNOS may result in low level of NO produced by MG-63 cells. This may imply a lower antibacterial activity for unencapsulated *E. faecalis* cps2 strain. However, since we studied only the mRNA expression of IL-1β gene, there may be other genes which induce discrepant expression iNOS mRNA values showed by different *E. faecalis* cps2 phenotypes. In addition, this study does not imply that the unencapsulated *E. faecalis* cps2 is not a virulent strain, but the lack of iNOS mRNA induction may be an indication of the tolerance immune response of osteoblast-like cells towards the internalized bacterium (unencapsulated *E. faecalis* cps2 strain).

## Conclusion

Regardless of the mechanism involved, this study demonstrated that adhesion and invasion to osteoblast-like cells do not solely rest on the expression of *E. faecalis* CPS. Capsule protects the detection of external proteins (PAMPs). In contrast, the absence of CPS, as shown by unencapsulated *E. faecalis* cps2, results in an increased of cells’ response. Therefore, this strain must find a way to survive by inducing the cells’ tolerance. The very mild iNOS induction may be a strategy to survive and protect the bacterium from host cells response. Thus, unlike the other tested strains, unencapsulated *E. faecalis* cps2 might not induce NO production. We speculated that the immune system response of MG-63 cells is reduced, and the low NO level is an index of cell tolerance to the bacterium. However, these mechanisms remain to be investigated at molecular level.

## Limitations

- We cannot exclude the possibility that the other unencapsulated *E. faecalis* strain (cps1 type) that was not included in this study, might show different effect in terms of host inflammation response.
- The encapsulated and unencapsulated *E. faecalis* cps2 are not isogenic strains. Therefore, we cannot explain what kind of influence of cps2 gene and CPS on the MG-63 cells’ response.

## Abbreviations

TLR-2: toll like receptor-2
IL-1β: Interleukin-1β
iNOS: inducible nitric oxide synthase
NO: nitric oxide
ATCC: American type culture collection
CFU: colony forming unit
DMEM: Dulbecco’s modified Eagles medium
IS6770: insertion sequence 6770
